# 3D-Printed Foods for Dysphagia: A Bibliometric Review

**DOI:** 10.3390/foods14122058

**Published:** 2025-06-11

**Authors:** Jinge Shao, Zhipeng Zheng, Jiamiao Hu, Natthida Sriboonvorakul, Shaoling Lin

**Affiliations:** 1 College of Food Science, Fujian Agriculture and Forestry University, Fuzhou 350002, China; 2 College of Life Sciences, University of Leicester, Leicester LE1 7RH, UK; 3 Department of Clinical Tropical Medicine, Faculty of Tropical Medicine, Mahidol University, Bangkok 10400, Thailand; 4 Integrated Scientific Research Base of Edible Fungi Processing and Comprehensive Utilization Technology, Fuzhou 350002, China

**Keywords:** 3D printing, dysphagia, bibliometric analysis

## Abstract

Dysphagia is a medical condition affecting millions globally. Traditional modified diets designed for individuals with dysphagia often focus primarily on improved swallowability, yet they typically fall short in terms of palatability, nutritional diversity, and visual appeal, leading to reduced food intake, malnutrition and reduced quality of life. Recent advancements in 3D-printing technology offer the potential to create texture-modified foods that not only facilitate swallowing but also preserve nutritional content and visual appeal. However, there is a noticeable gap in the comprehensive bibliometric analysis of studies on the use of 3D printing to address swallowing difficulties. To bridge this gap, this study systematically analyzes the literature on the development of 3D-printed foods tailored for individuals with dysphagia from the bibliometric perspective. The results highlight the top journals, leading countries, and prominent institutes/authors in this field. The study also examines the adoption of various 3D-printer brands, the key indicators used to assess the palatability of 3D-printed foods for dysphagia, and the common ingredients used for the development of 3D-printing ink. Overall, this review provides a comprehensive overview of current research trends in the development of 3D-printed food for dysphagia, offering valuable insights for future research in this area.

## 1. Introduction

In recent decades, dysphagia has emerged as a significant health concern, affecting a growing number of patients [1]. Dysphagia, defined as difficulty swallowing food, typically manifests as abnormal delays in the movement of food during the swallowing process, affecting both ingestion and digestion [2]. This condition is prevalent across all age groups, from neonates to the elderly [3]. One approach to mitigating the impact of dysphagia involves modifying the texture of solid foods and adjusting the consistency or viscosity of liquids. However, such modifications often result in pureed foods, which, while easier to swallow, may lack visual appeal [4,5]. Furthermore, nutrient loss during the processing of pureed foods is also a significant concern. Therefore, improving the texture, appearance, and nutrition of food has become a key challenge in addressing the dietary needs of dysphagia patients.

Three-dimensional food printing represents a cutting-edge food manufacturing technology that offers significant advantages, including customization and personalization, enhanced product functionality and innovation in food design [6]. With the rapid advancements in 3D-printing technology, researchers have increasingly applied it to the food industry, particularly for the production of dysphagia-friendly foods [7]. In particular, extrusion-based 3D printing of food materials has opened up new possibilities for addressing this issue, as the customized foods produced through this method can meet the daily dietary and nutritional needs of individuals with dysphagia [8]. This process enables the stacking of food layers to design products with tailored textures, flavors, shapes, and sizes [9]. Moreover, 3D-printed foods can be assessed using the International Dysphagia Diet Standardisation Initiative (IDDSI) guidelines to determine their suitability for dysphagia patients [10,11]. Currently, significant progress has been made in applying 3D printing to the field of dysphagia, with many publications focusing on developing suitable inks, and optimizing printing processes to enhance food quality and safety of printed foods.

Bibliometric analysis, a data-driven method for examining large volumes of academic publications, reveals relationships within a given field by providing statistical insights [12]. This may help researchers understand the research landscape in a certain field by highlighting emerging publication trends and identifying influential authors [13]. In the domain of personalized nutrition, bibliometric analysis has been employed to explore the functional properties of customized foods, such as their antioxidant, anti-inflammatory, antihypertensive, and antihyperglycemic effects [14,15,16,17]. However, a bibliometric analysis specifically focused on the application of 3D printing in the creation of dysphagia-friendly foods is still lacking.

This study aims to employ bibliometric tools to analyze and visualize the publications focusing on the use of 3D-printing technology in addressing dysphagia. Based on the analysis, a future perspective for the field of 3D printing for dysphagia is also proposed. The review may provide an overview of the current research landscape and development trends in this field, offering valuable guidance for future research directions.

## 2. Materials and Methods

### 2.1. Data Source, Search Strategy, and Selection Criteria

**Figure 1:**
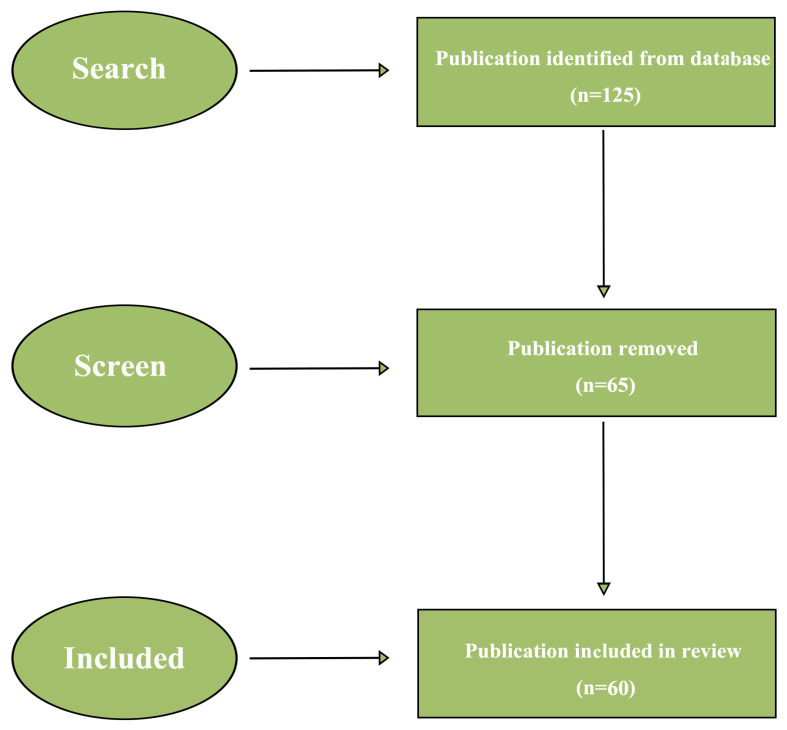
Flowchart of the search and selection process.

As shown in Figure 1, relevant publications were screened from the Science Citation Index Expanded (SCI-EXPANDED) within the Web of Science (WOS) database, utilizing the following search strategy: TS = (“3D printing”) and TS = (“dysphagia”) or TS = (“3D printing”) and TS = (“IDDSI”) or TS = (“3D printing”) and TS = (“texture-modified foods”) or TS = (“3D printing”) and TS = (“swallowing disorder”). The retrieved publications were up to the end of 2024, and publications other than “article” type were excluded. A total of 125 publications were initially retrieved, of which 60 were selected after a thorough review and screening process. Excluded publications mainly included those unrelated to the aim of this study, such as “3D printed polymer scaffolds used in non-food applications” [18].

### 2.2. Analysis Methods

To conduct a comprehensive analysis of the selected publications, VOSviewer (version 1.6.20) was used to extract bibliometric data, including publication year, country, institution, author, and journal, with visualizations being subsequently generated accordingly. To further enhance the visual clarity, Origin 2024 was employed for the creation of plots and diagrams.

## 3. Results

### 3.1. Publication Growth

**Figure 2:**
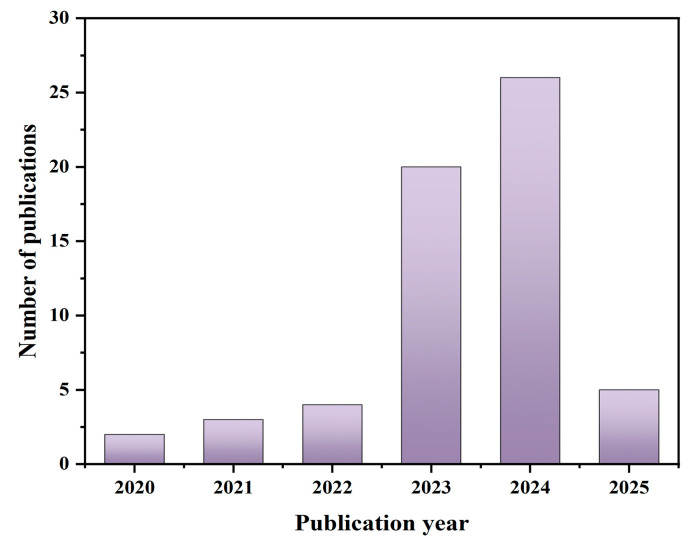
Annual trends of global publication outputs in the field.

As shown in Figure 2, a total of 60 publications investigating the applicability of 3D printing in creating tailored dysphagia foods have been identified since 2020, with a rapid rise in the number of publications from 2023 onwards. Notably, since some journals offer an “Early Access” option, several publications are categorized as being published in 2025, underscoring the fact that 3D printing for dysphagia foods remains a prominent area of research in 2025.

### 3.2. Leading Countries, Institutions and Authors

According to the search results, identified publications were authored by scholars in 20 countries. Table 1 presents the top 10 countries based on the volume of publications. Among these, China leads with the highest number of publications (38 publications), far surpassing other countries. This indicates that China has been actively conducting research in this area. Notably, while the United States and European countries often serve as leaders in many scientific domains, Australia and Canada have emerged as significant contributors in this specific field, ranking second and third, respectively. In particular, the 10 articles published in Australia received 408 citations, resulting in an obvious higher average citation count per publication. This suggests that Australian publications made considerable impacts and received significant attention from the research community.

**Figure 3:**
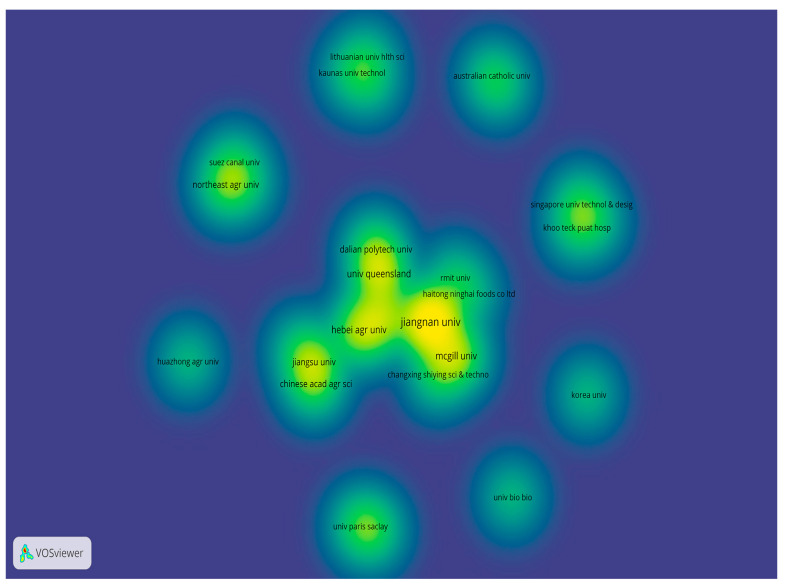
The density plot of collaborations between organizations. Note: each dot has a color: the higher the density, the closer the color is to yellow, and the lower the density, the closer the color is to blue.

A total of 89 organizations have contributed to the literature included for analysis. Table 2 lists the top five organizations based on their publication output. Jiangnan University leads with 13 publications, 376 citations, and a total link strength of 19, reflecting its significant research output and strong academic collaboration. McGill University, Hebei Agricultural University, and the University of Queensland have each published six publications, with the University of Queensland having 361 citations, followed by Hebei Agricultural University with 299 citations, and McGill University with 89 citations. As shown in the density plot of collaborations between organizations (Figure 3), the above top five institutions have also established strong collaborative partnerships (e.g., links between Jiangnan University and McGill University, Hebei Agricultural University and Shaanxi University of Science and Technology, etc.).

**Figure 4:**
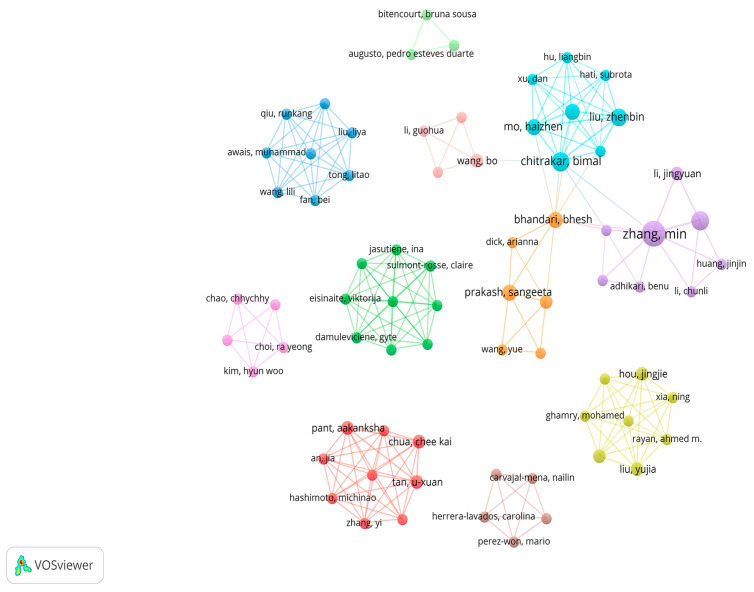
Author co-occurring network diagram. Note: a color represents a cluster, and a connecting line represents a cooperative relationship; the thicker the line, the closer the cooperation.

Next, the co-occurrence analysis was conducted for the authors who have published at least two publications within this area. As shown in Figure 4, a total of 272 scholars participated in research on the application of 3D printing in dysphagia foods, with Min Zhang being the most prolific author, having published 10 publications. In addition, the network diagram illustrating author–co-authorship relationships can be divided into 11 clusters, each of which may represent a research team. Among these, nine research teams have at least five members, and six research teams have at least eight members. This indicates that the research teams in this field typically exist on a scale of 5 to 10 scholars. The lines connecting the clusters represent collaboration between teams, which shows that fewer than half of the clusters presented are interconnected, suggesting less emphasis on broad collaboration among scholars in the field.

### 3.3. Brand of 3D-Printer Analysis

Among the publications analyzed, 32 publications utilized 3D printers manufactured by Shiyin Co. Ltd., Hangzhou, China; 7 used printers from Natural Machines, Barcelona, Spain; 4 employed printers from Wiiboox, Nanjing, China; and 3 publications utilized 3D printers from Hyrel, Norcross, GA, USA. Additionally, both Changxing Shiyin Technology Co. Ltd., Hangzhou, China, and PORIMY 3D Printing Technology Co., Ltd., Kunshan, China, were reported to be the manufacturers in two publications. This likely reflects the dominance of Chinese institutions in this research area and, to some extent, it also reflects the fact that Chinese food 3D printers are already comparable in quality to many European and American manufacturers.

### 3.4. Keywords Statistics

**Figure 5:**
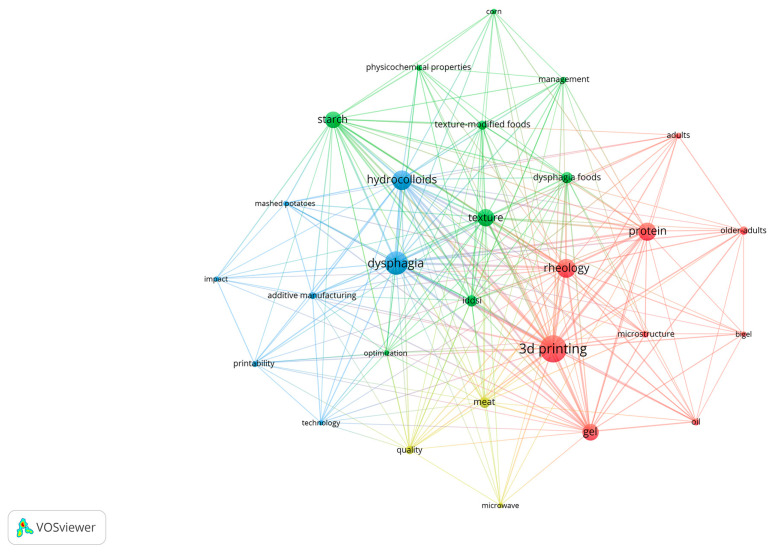
The co-occurrence network analysis of keywords. Note: a color represents a cluster, and a connecting line represents a cooperative relationship; the thicker the line, the closer the cooperation.

In the 60 included publications, a total of 223 keywords were extracted. From these, 28 keywords that appeared three times or more were selected for further clustering analysis, which may reflect the hotspots in this area [19]. As shown in Figure 5, “3D printing” is the most frequently occurring keyword, appearing 44 times, followed by “Dysphagia,” “Hydrocolloids,” “Rheology,” and “Protein,” which appeared 34, 25, 24, and 23 times, respectively.

Cluster I mainly consists of nine keywords, with “3D printing” being most mentioned (44 times), followed by common keywords like “rheology”, “ protein”, and “gel”. This cluster clearly indicates that the success of 3D printing in this context is highly dependent on the rheological properties of the printing inks [20]. For instance, if the viscosity of the printing ink is too low, it is easy for it to be extruded from the nozzle, but the printed product has poor support properties and is prone to collapse. Conversely, if the viscosity is too high, the ink may provide better structural support, but it tends to clog the nozzle and requires significantly higher extrusion pressure during the printing process. To meet these requirements, materials must demonstrate shear-thinning behavior, a characteristic well supported by gel systems.

Cluster II mainly consists of nine keywords related to texture, with “texture” being mentioned the most (21 times), followed by “starch”, “IDDSI” and “dysphagia foods” and “corn”. This suggests that texture is important for dysphagia foods, and the IDDSI provides a detailed method for measuring texture: the standards established by the International Dysphagia Diet Standardisation Initiative (IDDSI) are crucial for determining whether a food is suitable for individuals with dysphagia [21]. Starch and corns are very suitable ingredients for the development of foods that are difficult to swallow.

Cluster III mainly consists of keywords related to hydrocolloids, with a total of seven keywords. Among these, “dysphagia” is mentioned the most (34 times), followed by commonly used keywords such as “hydrocolloids” and “printability”, “additive manufacturing”. This suggests that additive manufacturing, also known as 3D printing, is an important solution to dysphagia. Hydrocolloids usually need to be added to the print material to improve the printability.

Cluster IV mainly consists of keywords related to microwave, with a total of three keywords, “microwave”, “meat” and “quality”. In the field of 3D printing for dysphagia, microwaves are used as both a pre-treatment and post-treatment method. For non-printable and hard substances such as meat, post-processing with microwaves can improve the texture, keeping it soft while maintaining a certain structure.

### 3.5. Journal Analysis and Citation Analysis

**Figure 6:**
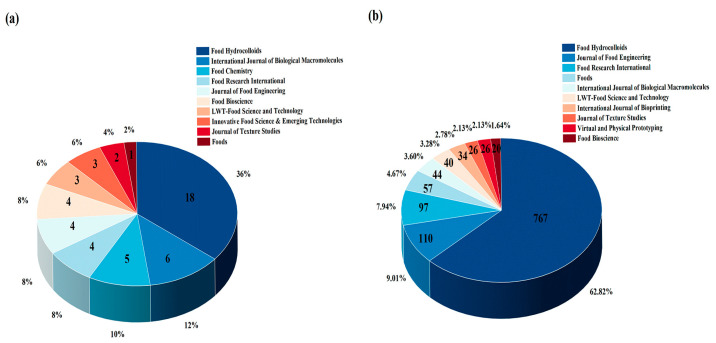
The main information regarding publications. (**a**) The major journals with the highest number of publications; (**b**) the most-cited major journals.

Figure 6 presents the leading journals in this field. *Food Hydrocolloids* ranks first with 18 publications, accounting for 36% of the total, followed by the *International Journal of Biological Macromolecules* with 6 publications (12%), *Food Chemistry* with 5 publications (10%), and *Food Research International*, *Journal of Food Engineering* and *Food Bioscience* with 4 publications each (8%). Similarly, analysis by citation counts also showed that *Food Hydrocolloids* again leads with 767 citations, representing 62.82% of the total, followed by *Journal of Food Engineering* with 110 citations (9.01%), *Food Research International* with 97 citations (7.94%), and *Foods* with 57 citations (4.67%). The high publication count and high citation count of *Food Hydrocolloids* reflected the fact that hydrocolloids are essential components in 3D-food printing, particularly for contributing to the printability, shape stability, and functionality of the printed products. In the Supplementary Materials, Table S1 lists the 10 most cited publications within this field. Interestingly, the publications in the *Food Hydrocolloids* journal also dominated this table, reinforcing the crucial role of hydrocolloids in developing tailored 3D-food printing inks for dysphagia-friendly foods. It is worth noting that among the included publications, all journals are categorized as Q1 or Q2 journals according to the Journal Citation Reports, except one article published in *Journal of Food Processing and Preservation*, indicating that the research conducted in this field is generally of high academic quality.

## 4. Discussion

### 4.1. Useful Indicators to Evaluate 3D-Printed Food Materials for Dysphagia

The International Dysphagia Diet Standardisation Initiative (IDDSI) has established a comprehensive framework to evaluate texture-modified foods and thickened liquids used in the management of dysphagia; thus, IDDSI testing methods are commonly used to determine the flow or textural characteristics of 3D-printed food, ensuring the food is appropriate for the specific needs of individuals with swallowing difficulties. IDDSI provides a globally standardized nomenclature and convenient tests to identify foods suitable for dysphagia [2]. Nearly all included publications (53 publications) adopted the IDDSI to assess whether a food can be classified as suitable for individuals with dysphagia (Table S2 in the Supplementary Materials [2,3,4,5,6,9,22,23,24,25,26,27,28,29,30,31,32,33,34,35,36,37,38,39,40,41,42,43,44,45,46,47,48,49,50,51,52,53,54,55,56,57,58,59,60,61,62,63,64,65,66,67,68]). Notably, the IDDSI framework does not specify testing equipment, and, as a result, it lacks defined numerical parameters for texture and rheological properties such as hardness, chewiness, and cohesiveness [43]. Meanwhile, IDDSI employs a qualitative assessment approach, classifying foods based on their flow and viscosity characteristics. However, the inherently subjective nature of this evaluation method may lead to variability among assessors, potentially impacting the consistency and accuracy of the classification [21].

In addition, a range of food-texture parameters are also widely used to evaluate the structural characteristics, processability, and mouthfeel of 3D-printed foods and their suitability for dysphagia patients. Key measurement indicators include rheological properties, texture, microstructure, and physical characteristics.

Rheological properties are critical in determining the feasibility of inks for 3D printing, influencing both extrusion capability and shape retention [25]. A total of 53 publications have reported using rheological parameters such as apparent viscosity (49 publications), elastic modulus (G′), viscous modulus (G″) and creep property to assess printability of food inks (Table S2 in the Supplementary Materials [2,3,5,6,9,20,22,23,24,25,26,27,28,29,30,31,32,33,34,35,36,37,38,39,40,41,42,43,45,46,47,48,49,50,51,53,54,55,57,58,59,60,61,63,64,65,66,67,68,69,70,71,72]). The phenomenon where apparent viscosity decreases with increasing shear rate is known as shear thinning. Shear-thinning behavior is not only suitable for 3D printing, but also beneficial for dysphagia foods [26], as it ensures that food remains structured at rest yet flows easily during swallowing [50]. Modulus and creep property reflect a material’s resistance to deformation. The intersection of elastic and viscous moduli, known as yield stress, is a critical parameter in 3D printing, as it defines the minimum force required for ink flow [26]. For dysphagia foods, yield stress determines the tongue force needed to move the food bolus. Additionally, creep reflects the time-dependent deformation characteristics of a material under constant stress, as well as its self-supporting performance. Through creep testing of a material under constant stress, its viscoelastic behavior can be obtained, thereby intuitively reflecting its swallowing characteristics [6,63]. Since 3D-printed samples must have both good shape support and extrusion ability, it is essential to balance extrusion ease and shape stability. Therefore, these parameters are widely measured for developing dysphagia foods using 3D-printing technology [63].

Texture profile analysis (TPA) is a widely used quantitative method for evaluating food texture. Among the reviewed studies, 52 assessed TPA parameters such as hardness (51 publications), cohesiveness (48 publications), adhesiveness (39 publications), and gumminess (24 publications), providing valuable insights into food texture and chewability (Table S2 in the Supplementary Materials [2,4,5,6,9,20,22,23,24,25,26,27,28,30,31,32,34,36,37,38,40,41,43,44,45,46,47,48,50,51,53,54,55,56,57,58,59,60,61,62,63,64,65,66,67,68,69,70,71,72,73,74]). Considering that TPA is a directly quantifiable index, it may be useful for assessing the suitability of 3D-printed food for dysphagia [21].

Water distribution and mobility significantly affect the structural and rheological properties of 3D-printed foods. Thus, Low-Field Nuclear Magnetic Resonance (LF-NMR), Fourier transform infrared spectroscopy (FTIR), and Scanning electron microscopy (SEM) are also commonly used analytical techniques for determining the suitability of 3D-printed food for dysphagia [23]. Specifically, LF-NMR, reported in 27 studies, assesses water state and mobility, while FTIR, utilized in 28 studies, provides molecular-level insights into hydrogen bonding and network structures, and SEM, reported in 35 studies, reveals the microstructural characteristics of ink formulations (Table S2 in the Supplementary Materials [5,9,20,22,23,24,25,27,29,33,34,35,37,38,40,41,43,44,46,47,48,50,51,53,55,57,58,59,60,61,63,64,65,69,72]).

Printability is another key criterion for evaluating 3D-printed food, ensuring smooth extrusion, high printing accuracy, and post-printing stability. Among the reviewed studies, 58 assessed printability by printing hollow cylinders or other shapes and evaluating smoothness, continuity, shape retention, and dimensional accuracy relative to design models (Table S2 in the Supplementary Materials [2,3,4,5,6,9,20,22,23,24,25,26,27,28,29,30,31,32,33,34,35,36,37,38,39,40,41,42,43,44,45,46,47,48,49,50,51,52,53,54,55,56,57,58,59,60,61,62,63,64,65,66,67,68,69,70,71,72]). High printability is essential for producing well-defined, stable 3D-printed food structures suitable for dysphagia management [31].

### 4.2. Analysis of Main Ingredients in the 3D-Print Ink

**Figure 7:**
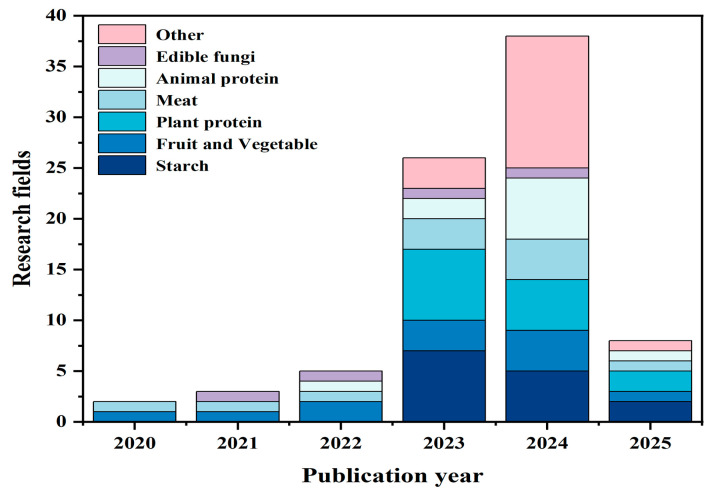
The proportions of different types of publications per year.

A wide range of food ingredients, including proteins, starches, and fats, can be used for food printing. To further explore the most commonly used ingredients in the development of 3D-printed foods for dysphagia, the included publications were also categorized based on the primary ingredient source. As shown in Figure 7 and Table S3 in the Supplementary Materials [2,3,4,5,6,9,20,22,23,24,25,26,27,28,29,30,31,32,33,34,35,36,37,38,39,40,41,42,43,44,45,46,47,48,49,50,51,52,53,54,55,56,57,58,59,60,61,62,63,64,65,66,67,68,69,70,71,72,73,74], plant-derived starches and proteins were the most extensively studied, each reported in 14 publications, followed by fruits and vegetables with 12 publications. Animal-derived meats were investigated in 11 publications, while animal proteins were examined in 10. Edible fungi were reported in four studies, whereas other ingredients (including beeswax, low-acyl gellan gum, egg yolk, carboxymethylcellulose, and corn oil) were explored in 17 publications.

The number of publications using plant-derived starch and proteins is the highest, with 14 publications each, making them the main research focus. Starch appears to be an ideal base material for 3D-printed foods, due to its viscoelastic properties upon gelatinization, ensuring precision and stability in 3D-printed structures [52]. This behavior enables the creation of complex shapes while maintaining structural integrity. Additionally, its shear-thinning behavior enhances extrusion and shape retention, while its natural biocompatibility and modifiability also makes it suitable for food applications. In addition, whole grain starch is rich in various micronutrients and phytochemicals, making it especially suitable for individuals with swallowing difficulties [63]. Notably, recent studies (three articles published in 2023) have also leveraged dual-nozzle 3D printing to optimize starch-based formulations for improved swallowability. Plant-derived protein, such as those from soy and peas, are also widely recognized for their positive impact on human health [75]. In particular, plant proteins offer sustainable, cost-effective, and hypoallergenic protein sources with high bioavailability for individuals with swallowing difficulties [25,29]. Thus, extensive studies (14 publications) have been performed to explore their incorporation into 3D-printed foods to help address malnutrition risks in dysphagic individuals.

Consuming an appropriate amount of fruits and vegetables is considered an effective way to prevent nutritional deficiencies for dysphagia patients [76]. Therefore, a considerable number of publications (12 publications) also included fruits and vegetables as the main ingredients to develop dysphagia-friendly foods using vegetables and fruits. However, due to their high water content and low carbohydrate and fat percentages, fruits and vegetables often pose great challenges in 3D printing [48]. Thus, to improve the 3D-printing performance of fruits and vegetables, hydrocolloids, such as xanthan gum and carrageenan, are commonly added simultaneously to enhance gel structure and printability (10 publications employed this strategy). Notably, one particularly noteworthy publication explored the utilization of vegetable waste (spinach stems and kale stalks [49]) as main ingredients for 3D printing. This innovative approach not only advances waste recycling, but also contributes to mitigating the environmental impact of food waste.

Meat, although providing all nine essential amino acids required by the human body, presents textural barriers for dysphagic individuals [73]. A total of 11 publications have investigated modifications to meat matrices through the incorporation of emulsions, gels, and hydrocolloids, aiming to enhance 3D printability while preserving essential amino acids. Specifically, three studies focused on pork, demonstrating that it can be successfully 3D printed using high internal phase Pickering emulsions and bigels, facilitating the development of dysphagia-friendly foods with reduced fat content [40] and enriched with functional compounds [73]. Similarly, three publications have focused on beef, primarily addressing post-printing treatments such as reheating [44], spray drying [45], and freeze-crosslinking [50]. These studies highlight the significant influence of additives (xanthan gum and guar gum [44], potato starch-based additives [45], and sodium alginate and calcium chloride crosslinking [50]) on the properties of 3D-printed beef. Additionally, four publications have explored the 3D printing of fish meat, with a particular emphasis on optimizing texture, taste, and nutritional composition to better accommodate the dietary requirements of individuals with dysphagia while enhancing the overall sensory experience.

In addition, animal protein (10 publications), in particular whey protein (4 publications), have also gained attention for their role in emulsions and gel-based structures, aiding in the formulation of dysphagia-friendly foods with improved nutritional value. In addition, the application of edible fungi (such as *Agaricus bisporus* [24], *Lentinus edodes* [26], *Hypsizygus marmoreus* by-products [28], and *Auricularia auricula* [32]) in 3D printing were also studied, mainly by scholars from Shaanxi University of Science and Technology (three of four available publications). These available publications mainly focus on improving printability through the addition of hydrophilic colloids, developing inks suitable for dysphagia diets.

Based on the analysis of the included publications, starch and plant proteins from plant sources, meat and animal proteins from animal sources, as well as fruits and vegetables and edible mushrooms, are the materials most commonly used for 3D printing for dysphagia.

## 5. Future Perspectives

While 3D-food printing has made substantial advancements in addressing the challenges associated with dysphagia, there remains considerable scope for further refinement, particularly in enhancing printing speed and precision. Currently, 3D printing for dysphagia primarily relies on single-nozzle extrusion, limiting structural complexity. The future will likely see increased adoption of dual-nozzle and coaxial 3D-printing technologies. These advancements allow for controlled layering of different ingredients, enabling intricate texture modifications that facilitate safer swallowing. Moreover, optimizing nozzle size and internal fill rates will refine food consistency, making it easier for dysphagia patients to ingest and digest.

Pre-processing techniques such as dry heating, fermentation, and twin-screw extrusion will also be promising approaches to enhance printability and nutritional properties. Post-processing methods, such as microwave heating, steaming, and freeze-drying, could be optimized to ensure that printed foods maintain their intended texture while preserving bioactive compounds. Such improvements could align printed foods more closely with the International Dysphagia Diet Standardization Initiative (IDDSI) framework, ensuring appropriate texture classifications.

In particular, 3D-printing technology represents a pivotal advancement in the preparation of personalized foods, offering precise control over ingredient composition to tailor nutritional content to individual needs. To further optimize dysphagia-friendly food formulations, future research must focus on understanding the relationship between ingredient composition and its physiological effects. This can be achieved through the development of diverse formulations that integrate multiple nutritional sources, ensuring a more comprehensive approach to personalized nutrition.

Moreover, while functional ingredients such as polyphenols and probiotics have been reported to be successfully incorporated into 3D-printed foods, their stability and bioavailability remain a challenge. Advanced analytical techniques, such as mass spectrometry, should be employed in future studies to validate the retention, release, and digestion of these bioactive compounds, ensuring that the intended nutritional benefits are preserved throughout the digestive process.

In addition, our analysis also revealed that the majority of studies did not include validation through human subjects. Indeed, only eight publications conducted sensory evaluations involving healthy individuals, while only studies assessed the suitability of the 3D-printed foods directly with dysphagia patients. Thus, in the future, it is recommended to include clinical trials to better translate the results into practical applications.

An analysis of the funding sources reported in the reviewed publications reveals that the vast majority of current research is supported by government-funded programs, reflecting a strong public-sector commitment to addressing the challenges associated with dysphagia. While this governmental support is commendable, the future development and commercialization of dysphagia-friendly foods would greatly benefit from the active involvement of private enterprises and venture capital. Such collaboration could accelerate the translation of research findings into practical, market-ready solutions that better serve individuals with dysphagia.

Last, but not least, to meet the increasing demand for tailored dysphagia-friendly foods, researchers must focus on developing more rapid and highly precise printing technologies. Additionally, the high cost of 3D-food printers continues to hinder their widespread adoption. Moving forward, efforts should be directed toward cost reduction, ensuring that these technologies become more accessible to dysphagia patients, thereby broadening their practical application and impact. In addition, food safety and hygiene standards should be not overlooked, since these are also critical considerations in the development of 3D-printed foods, particularly for vulnerable populations such as individuals with dysphagia. For instance, ensuring microbiological safety during printing and post-processing is essential, as improper handling or temperature control can lead to contamination. Therefore, regulatory bodies must establish clear guidelines to ensure that printed foods meet nutritional, textural, and safety requirements tailored to the needs of at-risk consumers.

## 6. Conclusions

This study employs bibliometric analysis to systematically assess and visualize the research landscape of 3D-food printing for dysphagia, based on publications indexed in the Web of Science (WOS) database up to the end of 2024. The findings indicate a substantial increase in research output over time, with 2024 marking the peak in publication activity. Notably, China, along with the Min Zhang’s research team, has emerged as the leading contributor in this domain, while *Food Hydrocolloids* stands as the most prolific journal publishing relevant studies.

This study analyzed several indicators such as IDDSI, rheological properties, texture, microstructure and physical properties for assessing 3D-printed food materials for dysphagia. Among them, IDDSI is the most widely used test metric to assess compliance with dysphagia diets. Immediately following the analysis of printing materials commonly used to develop food products for dysphagia using 3D printing, plant-derived starches and proteins were the most widely studied, followed by fruits and vegetables, animal-derived proteins, and animal meats. Edible mushrooms have also been included in studies, in small quantities. Since these materials are rich in nutrients but not texturally suitable for 3D printing or for dysphagia populations, hydrophilic colloids such as xanthan gum are often used to improve their printability for the development of dysphagia foods.

Finally, we propose future improvements to address the current challenges that arise in the field of 3D printing for dysphagia; for example, printing more complex products using dual-nozzle and coaxial 3D-printing techniques and the use of more advanced analytical techniques such as mass spectrometry to validate the retention, release and digestion of bioactive compounds such as polyphenols and probiotics.

In summary, this study provides a comprehensive synthesis of the current research landscape, elucidates the intricate relationship between 3D printing and dysphagia management, and offers valuable perspectives to guide future investigations in this evolving field.

## Figures and Tables

**Table 1:** Top ten countries ranked by publication output.

Country	Publication Count	Citations	Citations per Publication
China	38	780	20.5
Australia	10	408	40.8
Canada	9	132	14.7
France	5	31	6.2
India	4	193	48.3
Brazil	3	30	10.0
Singapore	3	368	122.7
Korea	3	74	24.7
Egypt	2	4	2.0
Lithuania	2	1	0.5

**Table 2:** Top five institutions ranked by publication output.

Organization	Publication Count	Citations	Total Link Strength
Jiangnan University	13	376	19
McGill University	6	89	9
Hebei Agricultural University	6	299	8
The University of Queensland	6	361	7
Shaanxi University of Science and Technology	5	288	7

## Data Availability

The original contributions presented in this study are included in the article/Supplementary Materials. Further inquiries can be directed to the corresponding authors.

## Supplementary Materials

The following supporting information can be downloaded at: https://www.mdpi.com/article/10.3390/foods14122058/s1, Table S1: The Top Cited Publications within this field.; Table S2: Main Ingredients, Assessment Indicators, and Photographs of Dysphagia-Friendly 3D-Printed Foods. Table S3: Main Ingredients in Printing Ink for Preparation of Dysphagia-Friendly 3D-Printed Foods.
