# Supplementary material for: 3D-Printed Foods for Dysphagia: A Bibliometric Review
Source: Foods. 2025 Jun 11;14(12):2058. doi: 10.3390/foods14122058 (PMC12191984; doi:10.3390/foods14122058)
Supplement: Supplementary file 1 [file foods-14-02058-s001.zip › foods-3662746-Supplementary Materials.pdf]

Supplementary Materials:

Table. S1 The Top Cited Publications within this field.

Number	Publication year	Title	Citations	Journal
1	2021	3D food printing of fresh vegetables using food hydrocolloids for dysphagic patients	218	*Food Hydrocolloids*
2	2020	Feasibility study of hydrocolloid incorporated 3D printed pork as dysphagia food	192	*Food Hydrocolloids*
3	2022	Development of black fungus-based 3D printed foods as dysphagia diet: Effect of gums incorporation	130	*Food Hydrocolloids*
4	2023	Pea protein-xanthan gum interaction driving the development of 3D printed dysphagia diet	71	*Food Hydrocolloids*
5	2023	Investigation of 3D printing of apple and edible rose blends as a dysphagia food	70	*Food Hydrocolloids*
6	2021	3D Printing of Shiitake Mushroom Incorporated with Gums as Dysphagia Diet	59	*Foods*
7	2022	Coaxial 3D printing of chicken surimi incorporated with mealworm protein isolate as texture-modified food for the elderly	56	*Journal of Food Engineering*
8	2023	Feasibility of hydrocolloid addition for 3D printing of Qingtuan with red bean filling as a dysphagia food	38	*Food Research International*
9	2023	Hydrophilic colloids (Konjac gum/Xanthan gum) in 3D printing of transitional food from fish paste	36	*Food Hydrocolloids*
10	2021	Three-Dimensional Printing of Food Foams Stabilized by Hydrocolloids for Hydration in Dysphagia	35	*International Journal of Bioprinting*

Table. S2 Main Ingredients, Assessment Indicators, and Photographs of Dysphagia-Friendly 3D-Printed Foods

TPA	Indicators	IDDSI level	Picture	Reference
·Whole potato flour	·Printability/·Rheological properties/·TPA/·FT-IR/·SEM/·LF-NMR/·IDDSI	Level 4, Level 5	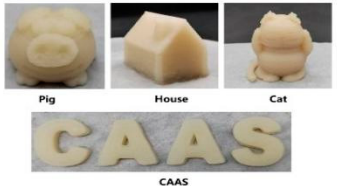	[1]
·Pea protein isolate/·Strawberry powder	·Printability·/Rheological properties/·TPA/·SEM/·LF-NMR/·IDDSI	Level 4	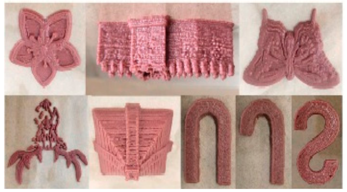	[2]
·*Agaricus bisporus*/·Soybean protein isolate	·Printability/·Rheological properties/·TPA/·FT-IR/·SEM/·LF-NMR/·IDDSI	Level 5	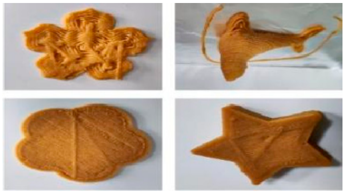	[3]

·Soy protein isolate powder /·Egg white protein powder/·Sunflower seed oil	·Printability/·Rheological properties/·TPA/·FT-IR/·SEM/·LF-NMR/·IDDSI	Level 5	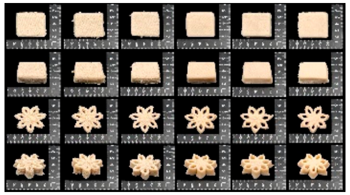	[4]
·*Lentinus edodes*/·Xanthan gum/·Arabic gum/·Kappa-carrageenan	·Printability/·Rheological properties/·TPA/·FT-IR/·LF-NMR/·IDDSI	Level 5	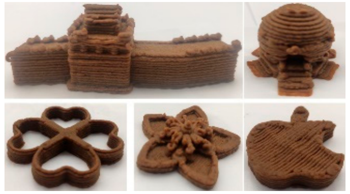	[5]
·Garden pea/·Leafy vegetables/·Potatoes/·Fish gelatin/·Xanthan gum	·Printability/·Rheological properties/·TPA/·FT-IR/·SEM/·IDDSI	Level 4	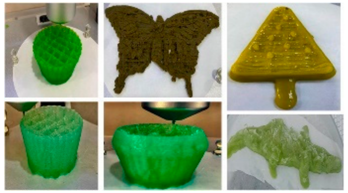	[6]
·*Hypsizygus marmoreus* by-products/·Calcium-enriched low-fat milk powder/·Xanthan gum/·Locust bean gum/·Kappa-carrageenan	·Printability/·Rheological properties/·TPA/·LF-NMR/·IDDSI	Level 5	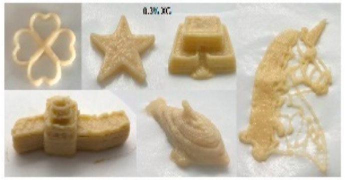	[7]

·Pea protein isolate/·Xanthan gum	·Printability/·Rheological properties/·FT-IR/·SEM/·LF-NMR/·IDDSI	Level 4	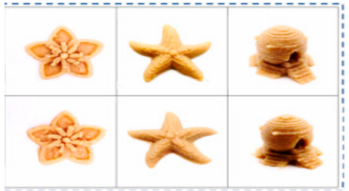	[8]
·Beeswax/·Low acyl gellan gum/·Algae oil	·Printability/·Rheological properties/·TPA/·FT-IR/·LF-NMR/·IDDSI	Level 4, level 5	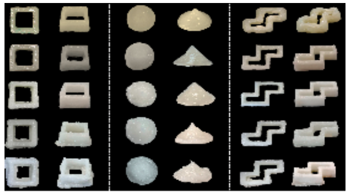	[9]
·Whey protein isolate/·Nanofibrils/·*Lactiplantibacillus plantarum*/·Glyceryl tributyrate/·High acyl gellan gum/·Fructooligosaccharides	·Printability/·Rheological properties/·TPA/·LF-NMR/·IDDSI	Level 5	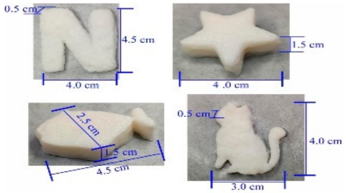	[10]
·*Auricularia auricula*/·Arabic gum/·Xanthan gum/·Kappa-carrageenan	·Printability/·Rheological properties/·TPA/·FT-IR/·LF-NMR/·IDDSI	Level 5	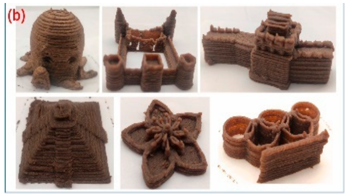	[11]

·Corn oil/·Low-acyl gellan gum/·Propylene glycol alginate/·Gliadin	·Printability/·Rheological properties/·FT-IR/·SEM/·LF-NMR/·IDDSI	Level 3-5	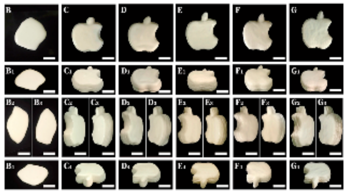	[12]
·Horse gram (*Macrotyloma uniflorum*) flour/·Chia seeds flour	·Printability/·Rheological properties/·TPA/·FT-IR/·SEM/·IDDSI	Level 6	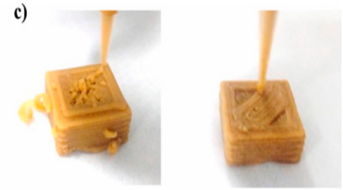	[13]
·Pea protein isolate/·Inulin/·Corn oil/·Sunflower seed oil/·Olive oil/·Flaxseed oil	·Printability/·Rheological properties/·SEM/·IDDSI	Level 4-5	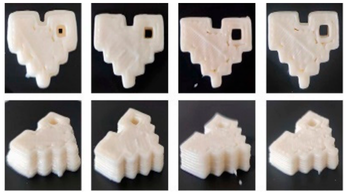	[14]
·Fresh garden peas /·Greek strained yoghurt /·Vegetable s tocks /·Extra virgin Greek olive oil /·Organic mint leaves powder/·Xanthan gum/·Guar gum /·Locust bean gum /·Arabic gum/·Kappa-carrageenan gum	·Printability/·Rheological properties/·TPA/·IDDSI	Level 4	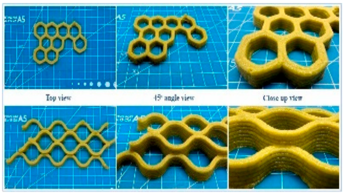	[15]

·Bean protein isolate/·Bovine gelatin/·Soy oil	·Printability/·Rheological properties/·TPA/·FT-IR/·SEM/·IDDSI	Level 5	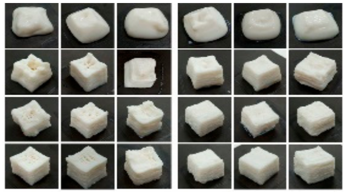	[16]
·White chub frozen surimi/·Deep-sea fish oil/·Transglutaminase/·Fish scale gelatin/·Xanthan gum	·Printability/·Rheological properties/·TPA/·SEM/·LF-NMR/·IDDSI	Level 6	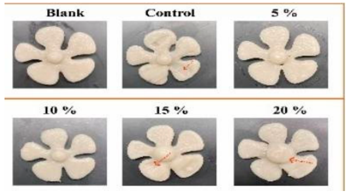	[17]
·Whey protein isolate/·Soybean oil/·Wheat/·Corn /·Rice /·Potato·/Sweet potato /·Cassava/·Mung bean/·Pea	·Printability/·Rheological properties/·TPA/·FT-IR/·SEM/·LF-NMR/·IDDSI	Level 5, Level 6	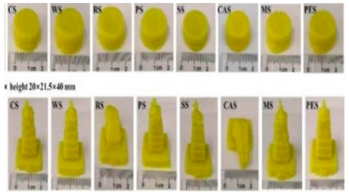	[18]
·Maize starch/·Orange juice/·Watermelon juice	·Printability/·TPA/·IDDSI	Level 5, Level 6	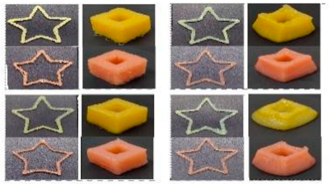	[19]

·Lean meat/·Bigel/·Meat broth/·Collagen/·Wheat fiber/·Psyllium Husk fiber/·Beetroot seasoning mixture/·Tomato seasoning mixture/·Meatballs seasoning mixture/·Glucose	·TPA	/	/	[20]
·Chicken egg yolk/·Carboxymethylcellulose/·Corn oil/·β-carotene	·Printability/·Rheological properties/·IDDSI	Level 4	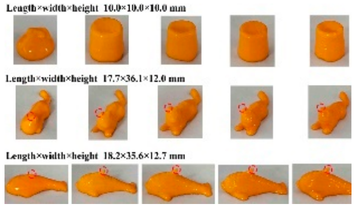	[21]
·Tenderloins/·High internal phase Pickering emulsions/·Tripolyphosphate/·NaCl	·Printability/·Rheological properties/·TPA/·SEM/·LF-NMR/·IDDSI	Level 5, Level 6	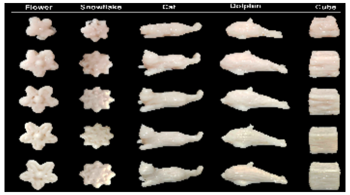	[22]

·Whey protein isolate/·Soybean oil/·NaCl/·Transglutaminase	·Printability/·Rheological properties/·TPA/·FT-IR/·SEM/·LF-NMR/·IDDSI	Level 5	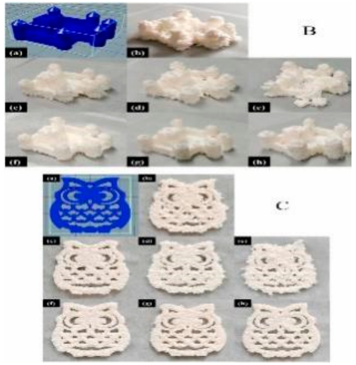	[23]
·Carboxymethylcellulose /·Egg yolk/·Corn oil /·β-Carotene	·Printability/·Rheological properties/·FT-IR/·IDDSI	Level 4	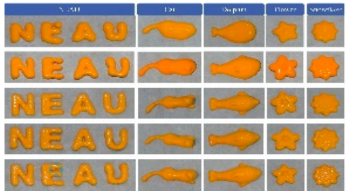	[24]
·Pork leg meat /·NaCl /·Xanthan gum /·Guar gum	·Printability/·Rheological properties/·TPA/·SEM/·IDDSI	Transitional foods	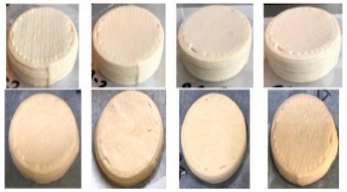	[25]

·Australian beef blade /·Sodium chloride /·Xanthan gum /·Guar gum	·Printability/·TPA/·SEM/·IDDSI	Level 4	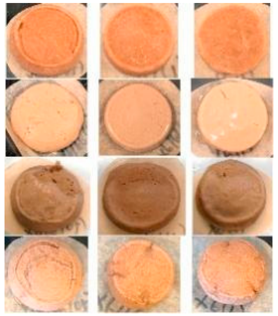	[26]
·Potato Starch/·NaCl /·Beef /·Gelatin	·Printability/·Rheological properties/·TPA/·IDDSI	Level 6	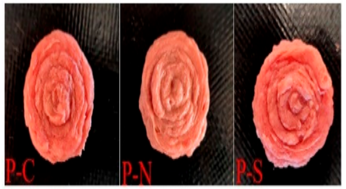	[27]
·Frozen surimi of silver carp/·Glycerol/·Transglutaminase	·Printability/·Rheological properties/·TPA/·SEM/·LF-NMR/·IDDSI	Transitional foods	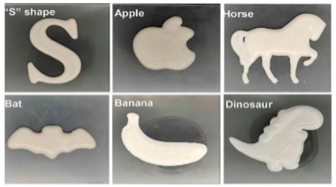	[28]

·Rice/·Black rice/·Oats/·Frozen silver carp surimi	·Printability/·Rheological properties/·TPA/·SEM/·IDDSI	Level 5	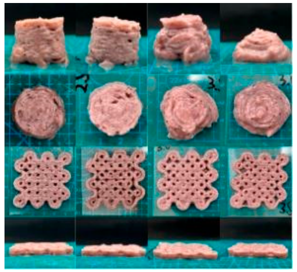	[29]
·Frozen hybrid sturgeon/·Konjac gum/·Xanthan gum	·Printability/·Rheological properties/·TPA/·FT-IR/·SEM/·IDDSI	Transitional foods	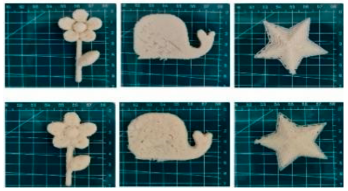	[30]
·Garden pea/·Carrot/·Bok choy/·Xanthan gum/·Kappa-carrageenan/·Locust bean gum	·Printability/·Rheological properties/·TPA/·SEM/·IDDSI	Transitional foods	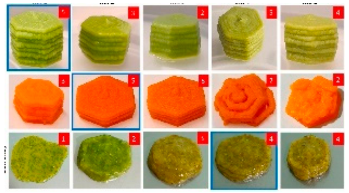	[31]
·Carrots/·Zucchini/·Olive oil/·Lentil protein concentrate ·*Smooth Chlorella*/·*Honey Chlorella*/·*White Chlorella*/·Xanthan gum	·Printability/·Rheological properties/·TPA/·SEM	/	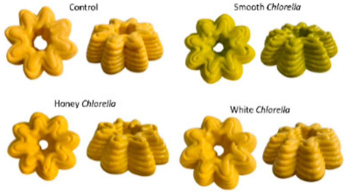	[32]

·Organic pureed carrots/·Gelatin/·Guar gum/·Xanthan gum	·TPA	/	/	[33]
·Spinach stems/·Kale stalks/·Pumpkin/·Potatoes/·Sweet potatoes/·Lemon zest/·Herbs cream cheese/·Cincalok chili/·Honey/·Mushrooms/·Onions/·Stir-fried tom yum paste/·Agar and gelatin hydrocolloids/·Spices	·Printability/·Rheological properties/·IDDSI	Transitional foods	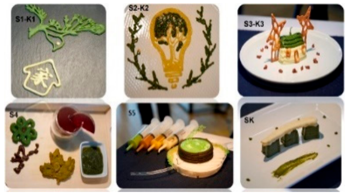	[34]
·Whole grain oat/·Chickpea and soybean flours/·α-glucan	·Printability/·Rheological properties/·FT-IR/·IDDSI	Level 5	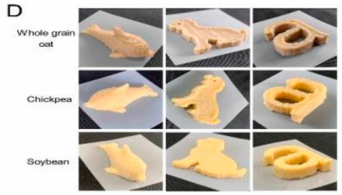	[35]
·Sodium alginate/·Ground beef	·Printability/·Rheological properties/·TPA/·SEM/·IDDSI	Level 4,<br>Level 5, Level 6	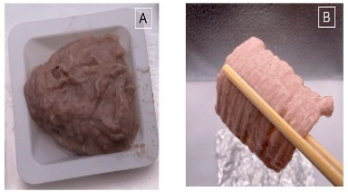	[36]

·Rice starch/·Curdlan/·NaCl	·Printability/·Rheological properties/·TPA/·FT-IR/·SEM/·LF-NMR/·IDDSI	Level 5	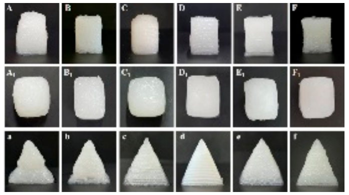	[37]
·Cassava starch/·Kappa-carrageenan/·Skim powdered milk	·Printability/·IDDSI	Level 5, Level 6	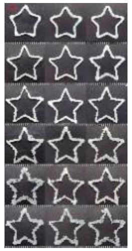	[38]
·Soy protein isolate/·Red cabbage/·Glycerol/·Sodium alginate	·Printability/·Rheological properties/·TPA/·FT-IR/·SEM/·IDDSI	Transitional foods	/	[39]
·Gelatin/·Whey protein isolate/·Xanthan gum/·Cellulose nanocrystals	·Printability/·Rheological properties/·TPA/·IDDSI	Level 5	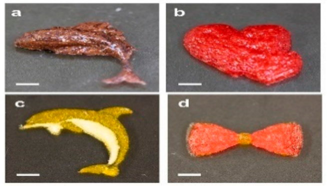	[40]

·Pea protein isolate/·Xanthan gum/·Konjac gum/·Locust bean gum	·Printability/·Rheological properties/·TPA/·FT-IR/·SEM/·LF-NMR/·IDDSI	Level 4	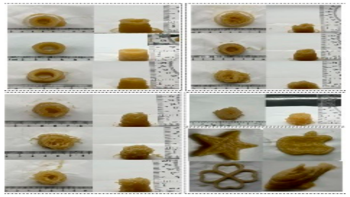	[41]
·Tofu/·Soy protein isolate/·Wheat fibre/·Psyllium husk powder/·Sugar/·Cocoa/·Citrus fresh liquid seasoning/·Bigel	·Printability/·TPA/·IDDSI	Level 6	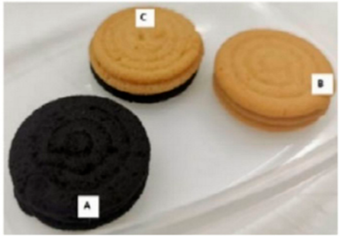	[42]
·*Tremella polysaccharides*/·Psyllium husk powder/·Soy protein isolate/·NaCl	·Printability/·Rheological properties/·TPA/·SEM/·LF-NMR/·IDDSI	Level 5	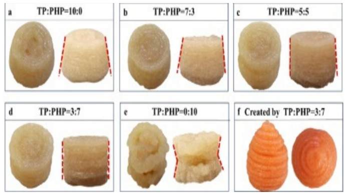	[43]
·Soy protein isolate/·Beeswax/·Corn oil/·Powder of curcumin/·β-carotene/·Betanin	·Printability/·Rheological properties/·TPA/·SEM/·LF-NMR/·IDDSI	Transitional food	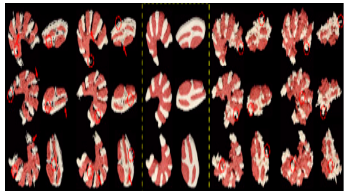	[44]

·Ginseng/·Isolated pea protein/·Stevia powder/·Black sepia ink powder/·Red beet extract powder/·Red ginseng extract	·Printability/·Rheological properties/·TPA/·FT-IR/·SEM/·IDDSI	Level 5	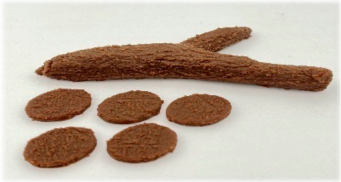	[45]
·Salmon protein isolate/·Corn starch	·Printability/·Rheological properties/·TPA/·FT-IR/·SEM/·IDDSI	Level 5	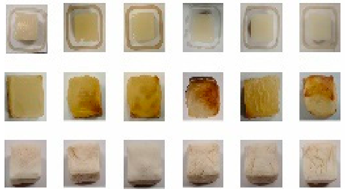	[46]
·Egg yolk powder/·Potato starch/·Corn Starch	·Printability/·Rheological properties/·TPA/·FT-IR/·SEM/·IDDSI	Level 4, Level 5	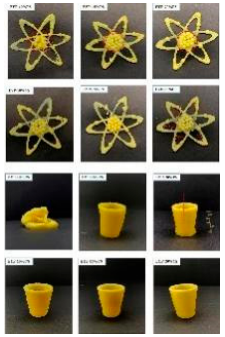	[47]

·Chickpea protein isolate flour/·Mealworm protein isolate powder/·Potato starch/·Alginate hydrogel	·Printability/·Rheological properties/·TPA/·SEM	/	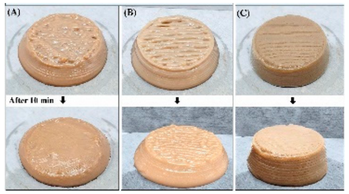	[48]
·Gelatin/·Regular native cassava starch/·Pasteurized whole grape juice	·Printability/·TPA/·IDDSI	Level 6	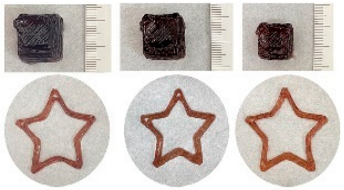	[49]
·Buckwheat flour/·Corn flour/·Chickpea protein isolate	·Printability/·Rheological properties/·TPA/·FT-IR/·SEM/·LF-NMR/·IDDSI	Level 5	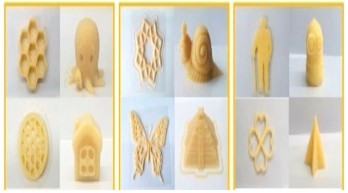	[50]
·Freeze-dried apple powder/·Edible rose powder/·Xanthan gum/·Basil seed gum	·Printability/·Rheological properties/·TPA/·FT-IR/·SEM/·LF-NMR/·IDDSI	Level 5	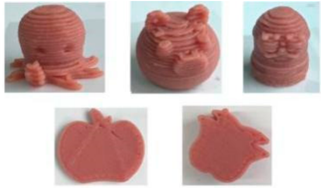	[51]

·Glutinous rice flour/·Wheat flour/·Wheat seedling powder/·Soluble soybean polysaccharide/·Red bean paste	·Printability/·Rheological properties/·TPA/·LF-NMR/·IDDSI	Level 5	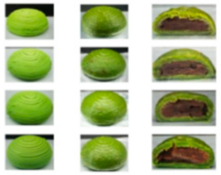	[52]
·Mung bean protein/·Rose powder/·Flaxseed gum	·Printability/·Rheological properties/·TPA/·FT-IR/·SEM/·LF-NMR/·IDDSI	Level 4	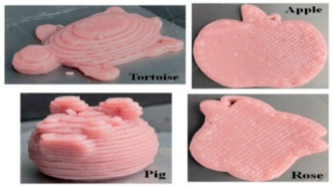	[53]
·Corn flour/·Fenugreek gum/·Flaxseed protein	·Printability/·Rheological properties/·TPA/·FT-IR/·LF-NMR/·IDDSI	Level 5	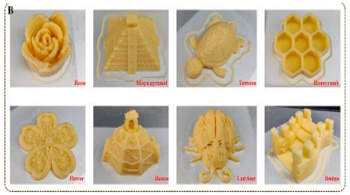	[54]
·Wheat core powder/·Potato snowflake whole powder/·Arabic Gum/·Red bean paste/·Soy oil	·Printability/·Rheological properties/·TPA/·IDDSI	Level 4	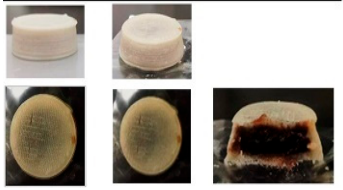	[55]

·Pumpkin powder/·Surimi	·Printability/·Rheological properties/·TPA/·LF-NMR	/	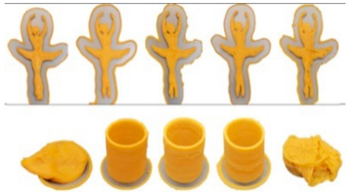	[56]
·Bean paste/·Wheat flour/·Rice flour/·Buckwheat powder	·Printability/·Rheological properties/·TPA	/	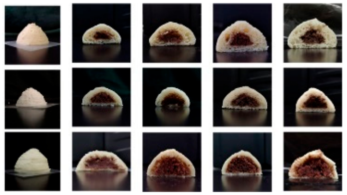	[57]
·White quinoa powder/·Brown rice powder/·Osmanthus Powder	·Printability/·Rheological properties/·TPA/·FT-IR/·LF-NMR/·IDDSI	Level 4	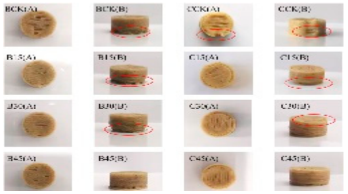	[58]
·Brown rice flour/·Milk/·*Lactobacillus bulgaricus*/·*Bifidobacterium longum*/·*Lactobacillus paracasei*, *Streptococcus thermophilus*/·*Lactiplantibacillus plantarum*	·Printability/·Rheological properties/·TPA/·FT-IR/·IDDSI	Level 4	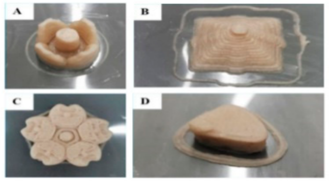	[59]

·Chicken surimi/·Mealworm protein isolate	·Printability/·Rheological properties/·TPA/·SEM	/	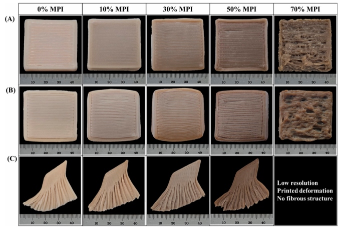	[60]

Table. S3 Main Ingredients in Printing Ink for Preparation of Dysphagia-Friendly 3D-Printed Foods.

Studies	Main ingredients	Categorization of main ingredients						
		Plant-derived			Animal-derived		Fungi-derived	
		Starch	Fruit and Vegetable	Plant protein	Meat	Animal protein	Edible fungi	Other
[1]	·Whole potato flour	+						
[2]	·Pea protein isolate<br>·Strawberry powder		+	+				
[3]	·*Agaricus bisporus*						+	
[4]	·Soy protein isolate powder<br>·Egg white protein powder			+		+		
[5]	·*Lentinus edodes*						+	
[6]	·Garden pea<br>·Leafy vegetables<br>·Potatoes<br>·Fish gelatin		+			+		
[7]	·*Hypsizygus marmoreus* by-products						+	
[8]	·Pea protein isolate			+				
[9]	·Beeswax<br>·Low acyl gellan gum							+
[10]	·Whey protein isolate					+		+

[11]	·Nanofibrils<br>·Glyceryl tributyrate<br>·*Lactiplantibacillus plantarum*<br>·*Auricularia auricula*			+	
[12]	·Gliadin<br>·Low-acyl gellan gum<br>·Propylene glycol alginate<br>·Corn oil		+		+
[13]	·Horse gram (*Macrotyloma uniflorum*) flour<br>·Chia seeds flour				+
[14]	·Pea protein isolate		+		
[15]	·Fresh garden peas<br>·Greek strained yoghurt<br>·Extra virgin Greek olive oil<br>·Vegetable stocks<br>·Organic mint leaves powder	+			+
[16]	·Bean protein isolate		+		

[17]	·White chub frozen surimi				+			
[18]	·Whey protein isolate<br>·Wheat<br>·Corn<br>·Rice<br>·Potato<br>·Sweet potato<br>·Cassava<br>·Mung bean<br>·Pea	+				+		
[19]	·Maize starch<br>·Orange and watermelon juice	+	+					
[20]	·Collagen<br>·Monoglycerides<br>·Diglycerides<br>·Lean meat<br>·Meat broth				+	+		+
[21]	·Egg yolk<br>·Carboxymethylcellulose<br>·Corn oi							+
[22]	·Tenderloins				+			

[23]	·Whey protein isolate<br>·Soybean oil			+	+
[24]	·Carboxymethylcellulose<br>·Egg yolk<br>·Corn oil				+
[25]	·Pork leg meat		+		
[26]	·Australian beef blade		+		
[27]	·Beef		+		
[28]	·Frozen surimi of silver<br>·Carp		+		
[29]	·Rice<br>·Black rice<br>·Oats<br>·Silver carp surimi	+	+		
[30]	·Frozen hybrid sturgeon		+		

[31]	·Garden pea<br>·Carrot<br>·Bok choy		+				
[32]	·Carrots<br>·Zucchini		+				
[33]	·Organic pureed carrots		+				
[34]	·Spinach stems<br>·Kale stalks		+				
[35]	·Whole grain oat<br>·Chickpea<br>·Soybean flours	+					+
[36]	·Ground beef				+		
[37]	·Rice starch	+					
[38]	·Milk						+
[39]	·Soy protein isolate			+			
[40]	·Gelatin<br>·Whey protein isolate<br>·Xanthan gum					+	+
[41]	·Pea protein isolate			+			
[42]	·Tofu			+			
[43]	·Soy protein isolate			+			

[44]	·Soy protein isolate<br>·Beeswax			+		+
[45]	·Ginseng					+
[46]	·Corn starch<br>·Salmon protein isolate	+			+	
[47]	·Egg yolk powder					+
[48]	·Chickpea protein isolate flour<br>·Mealworm protein isolate powder			+	+	
[49]	·Pasteurized whole grape juice		+			
[50]	·Buckwheat flour<br>·Corn flour	+				+
[51]	·Freeze- dried apple powder<br>·Edible rose powder		+			
[52]	·Glutinous rice flour<br>·Wheat flour<br>·Wheat seedling flour	+				
[53]	·Mung bean protein<br>·Rose powder		+	+		
[54]	·Flaxseed protein<br>·Corn flour	+		+		

[55]	·Wheat core powder	+				
[56]	·Pumpkin powder		+			
[57]	·Wheat flour	+				
[58]	·White quinoa powder<br>·Brown rice powder	+				+
[59]	·Brown rice flour	+				
[60]	·Chicken surimi<br>·Mealworm protein isolate			+	+	

Note: The "+" indicates that the study in this publication selected this primary material. Multiple "+" appearing in a publication indicate that the study chose several primary materials to be used together.

References

1. Wang, Y.; Zhao, R.; Liu, W.; Zhao, R.; Liu, Q.; Hu, H. Effect of twin-screw extrusion pretreatment on starch structure, rheological properties and 3D printing accuracy of whole potato flour and its application in dysphagia diets. *Int. J. Biol. Macromol.* **2024**, *278*, 134796. https://doi.org/10.1016/j.ijbiomac.2024.134796.
2. Xu, B.; Wang, X.; Chitrakar, B.; Xu, Y.; Wei, B.; Wang, B.; Lin, L.; Guo, Z.; Zhou, C.; Ma, H. Z. Effect of various physical modifications of protein isolate (PPI) on 3D printing behavior and dysphagia properties of strawberry-PPI gels. *Food Hydrocoll.* **2025**, *158*, 110498. https://doi.org/10.1016/j.foodhyd.2024.110498.
3. Xiao, K.; Zhang, J.; Pan, L.; Tu, K. Investigation of 3D printing product of powder-based white mushroom incorporated with soybean protein isolate as dysphagia diet. *Food Res. Int.* **2024**, *175*, 113760. https://doi.org/10.1016/j.foodres.2023.113760.
4. Zhao, P.; Kou, D.; Qiu, R.; Li, S.; Awais, M.; Tong, L.; Wang, L.; Fan, B.; Wang, F.; Liu, L. Development of soy protein emulsion gels-based 3D printed dysphagia foods: Effects of the egg white protein supplementation. *Food Hydrocoll.* **2025**, *160*, 110737. https://doi.org/10.1016/j.foodhyd.2024.110737.
5. Liu, Z.; Bhandari, B.; Guo, C.; Zheng, W.; Cao, S.; Lu, H.; Mo, H.; Li, H. 3D Printing of Shiitake Mushroom Incorporated with Gums as Dysphagia Diet. *Foods* **2021**, *10*, 2189. https://doi.org/10.3390/foods10092189.
6. Hassan, I.; Gani, A.; Mudabir, S. Development and characterization of 3D printed fish gelatin and vegetable blends as nutritious meal for dysphagic patients. *Food Hydrocoll.* **2024**, *157*, 110478. https://doi.org/10.1016/j.foodhyd.2024.110478.
7. Liu, Z.; Xing, X.; Mo, H.; Xu, D.; Hu, L.; Li, H.; Chitrakar, B. 3D printed dysphagia diet designed from Hypsizygus marmoreus by-products with various polysaccharides. *J. Food Eng.* **2023**, *343*, 111395. https://doi.org/10.1016/j.jfoodeng.2022.111395.
8. Liu, Z.; Chen, X.; Dai, Q.; Xu, D.; Hu, L.; Li, H.; Hati, S.; Chitrakar, B.; Yao, L; Mo, H. Pea protein-xanthan gum interaction driving the development of 3D printed dysphagia diet. *Food Hydrocoll.* **2023**, *139*, 108497. https://doi.org/10.1016/j.foodhyd.2023.108497.
9. Chao, E.; Yan, X.; Fan, L. Fabrication of edible inks for 3D printing as a dysphagia food: An emerging application of bigels. *Food Hydrocoll.* **2024**, *157*, 110463. https://doi.org/10.1016/j.foodhyd.2024.110463.
10. Zhang, Y.; Wang, Y.; Dai, X.; Li, Y.; Jiang, B.; Li, D.; Liu, C.; Feng, Z. Biointerfacial supramolecular self-assembly of whey protein isolate nanofibrils on probiotic surface to enhance survival and application to 3D printing dysphagia foods. *Food Chem.* **2024**, *460*, 140720. https://doi.org/10.1111/jfbc.13394.
11. Xing, X.; Chitrakar, B.; Hati, S.; Xie, S.; Li, H.; Li, C.; Liu, Z.; Mo, H. Development of black fungus-based 3D printed foods as dysphagia diet: Effect of gums incorporation. *Food Hydrocoll.* **2022**, *123*, 107173. https://doi.org/10.1016/j.foodhyd.2021.107173.
12. Hou, Y.; Sun, Y.; Zhang, P.; Wang, H.; Tan, M. Development and characterization of emulsion gels prepared via gliadin-based colloidal particles and gellan gum with tunable rheological properties for 3D printed dysphagia diet. *Int. J. Biol. Macromol.* **2023**, *253*, 126839. https://doi.org/10.1016/j.ijbiomac.2023.126839.
13. Satheeshkanth, S.;Bareen, M.; Joshi, S.; K. Sahu, J.; Naik, S. Engineering plant-based protein-rich soft and bite-sized 3D printed dysphagia food. *Int. J. Gastron. Food Sci.* **2024**, *37*, 100975. https://doi.org/10.1016/j.ijgfs.2024.100975.
14. Wang, Y.; Aluko, R.; McClements, D.; Yu, Y.; Xu, X.; Sun, Q.; Wang, Q.; Jiao, B.; Dai, L. Emulsion gel-based inks for 3D printing of foods for dysphagia patients: High internal type emulsion gel-biopolymer systems. *Food Hydrocoll.* **2024**, *156*, 110340. https://doi.org/10.1016/j.foodhyd.2024.110340.
15. Ekonomou, S.; Hadnadev, M.; Gioxari, A.; Abosede, O.; Soe, S.; Stratakos, A. Advancing dysphagia-oriented multi-ingredient meal development: Optimising hydrocolloid incorporation in 3D printed nutritious meals. *Food Hydrocoll.* **2024**, *147*, 109300. https://doi.org/10.1016/j.foodhyd.2023.109300.
16. Herrera-Lavados, C.; Tabilo-Munizaga, G.; Rivera-Tobar, D.; Carvajal-Mena, N.; Palma-Acevedo, A.; Moreno-Osorio, L.; Pérez - Won, M. Development of bean-based emulgels for 3D printing

applications: Feasibility for dysphagia diets. *J. Food Eng.* **2023**, *358*, 111687. https://doi.org/10.1016/j.jfoodeng.2023.111687.
17. Liang, W.; Wang, Y.; Li, C.; Wang, P.; Rong, J.; Liu, R.; Xiong, S.; Hu, Y. Development of easy-to-swallow and lipid-enhanced 3D printed surimi based on high internal phase emulsions. *Food Hydrocoll.* **2024**, *155*, 110207. https://doi.org/10.1016/j.foodhyd.2024.110207.
18. Wang, Z.; Chen, F.; Deng, Y.; Tang, X.; Li, P.; Zhao, Z.; Zhang, M.; Liu, G. Texture characterization of 3D printed fibrous whey protein-starch composite emulsion gels as dysphagia food: A comparative study on starch type. *Food Chem.* **2024**, *458*, 140302. https://doi.org/10.1016/j.foodchem.2024.140302.
19. Guedes, J.; Bitencourt, B.; Augusto, P. Modification of maize starch by dry heating treatment (DHT) and its use as gelling ingredients in fruit-based 3D-printed food for dysphagic people. *Food Biosci.* **2023**, *56*, 103310. https://doi.org/10.1016/j.fbio.2023.103310.
20. Kurapkienė, A.; Vinauskienė, R.; Jasutienė, I.; Keršienė, M.; Damulevičienė, G.; Knašienė, J.; Lesauskaitė, V.; Sulmont - Rossé, C.; Eisinaitė, V.; Leskauskaitė, D. Bigel as a curcumin delivery system and its application in 3D-printed in-between-meal foods to boost the immune system of elderly people. *Food Biosci.* **2024**, *61*, 104789. https://doi.org/10.1016/j.fbio.2024.104789.
21. Hou, J.; Tan, G.; Wei, A.; Gao, S.; Zhang, H.; Zhang, W.; Liu, Y.; Zhao, R.; Ma, Y. Carboxymethylcellulose-induced depletion attraction to stabilize high internal phase Pickering emulsions for the elderly: 3D printing and β-carotene delivery. *Food Chem.* **2024**, *447*, 139028. https://doi.org/10.1016/j.foodchem.2024.139028.
22. Hou, J.; Liu, M.; Liu, Y.; Chuang, R.; Zhang, H.; Zheng, L.; Li, H.; Xia, N.; Ma, Y.; Rayan, A.; et al. Strategy to kill two birds with one stone: High internal phase Pickering emulsions to modulate 3D printed pork texture as a dysphagia diet. *Food Chem.* **2023**, *463*, 141319. *https://doi.org/*10.1016/j.foodchem.2024.141319.
23. Li, G.; Wang, B.; Lv, W.; Mu, R.; Zhong, Y. Effect of induction mode on 3D printing characteristics of whey protein isolate emulsion gel. *Food Hydrocoll.* **2024**, *146*, 109255. https://doi.org/10.1016/j.foodhyd.2023.109255.
24. Hou, J.; Liu, Y.; Ma, Y.; Zhang, H.; Xia, N.; Li, H.; Wang, Z.; Rayan, A.; Ghamry, M.; Mohamed, T. High internal phase Pickering emulsions stabilized by egg yolk-carboxymethylcellulose as an age-friendly dysphagia food: Tracking the dynamic transition from co-solubility to coacervates. *Carbohydr. Polym.* **2024**, *342*, 122430. https://doi.org/10.1016/j.carbpol.2024.122430.
25. Dick, A.; Bhandari, B.; Dong, X.; Prakash, S. Feasibility study of hydrocolloid incorporated 3D printed pork as dysphagia food. *Food Hydrocoll.* **2020**, *107*, 105940. https://doi.org/10.1016/j.foodhyd.2020.105940.
26. Dick, A.; Bhandari, B.; Prakash, S. Effect of reheating method on the post-processing characterisation of 3D printed meat products for dysphagia patients. *LWT* **2021**, *150*, 111915. https://doi.org/10.1016/j.lwt.2021.111915.
27. Liu, P.; Zhou, Z.; Zhou, W.; Liu, C.; Wang, N.; Zhang, F. Spray-Drying Assisted 3D Printing to Manufacture Beef Products. *Starch-Stärke* **2024**, *76*, 300286. https://doi.org/10.1002/star.202300286.
28. Zhu, J.; Cheng, Y.; Ouyang, Z.; Yang, Y.; Ma, L.; Wang, H.; Zhang, Y. 3D printing surimi enhanced by surface crosslinking based on dry-spraying transglutaminase, and its application in dysphagia diets. *Food Hydrocoll.* **2023**, *140*, 108600. https://doi.org/10.1016/j.foodhyd.2023.108600.
29. Li, C.; Yu, X.; Wang, Y.; Prakash, S.; Dong, X. Innovative 3D printed multigrain rice and silver carp surimi diets: Rheological, textural, and nutritional solutions for the elderly with dysphagia. *Food Biosci.* **2024**, *62*, 105314. https://doi.org/10.1016/j.fbio.2024.105314.
30. Yu, X.; Wang, Y.; Zhao, W.; Li, S.; Pan, J.; Prakash, S.; Dong, X. Hydrophilic colloids (Konjac gum/Xanthan gum) in 3D printing of transitional food from fish paste. *Food Hydrocoll.* **2023**, *137*, 108333. https://doi:org/10.1016/j.foodhyd.2022.108333.
31. Pant, A.; Lee, A.; Karyappa, R.; Lee, C.; An, J.; Hashimoto, M.; Tan, U.; Wong, G.; Chua, C.; Zhang, Y. 3D food printing of fresh vegetables using food hydrocolloids for dysphagic patients. *Food Hydrocoll.* **2021**, *114*, 106546. https://doi.org/10.1016/j.foodhyd.2020.106546.
32. Giura, L.; Urtasun, L.; Ansorena, D.; Astiasaran, I.; Raymundo, A. Printable formulations of protein and Chlorella vulgaris enriched vegetable puree for dysphagia diet. *Algal Res.* **2024**, *79*, 103447. https://doi.org/10.1016/j.algal.2024.103447.
33. Strother, H.; Moss, R.; McSweeney, M. Comparison of 3D printed and moulded carrots produced with gelatin, guar gum and xanthan gum. *J. Texture Stud.* **2020**, *51*, 852–860. https://doi.org/10.1111/jtxs.12545.
34. Pant, A.; Leam, P.; Chua, C.; Tan, U. Valorisation of vegetable food waste utilising three-dimensional food printing. *Virtual Phys. Prototyp.* **2023**, *18*, e2146593. https://doi.org/10.1080/17452759.2022.2146593.

35. Dai, Y.; Liu, Y.; Wang, Z.; Xu, W.; Dong, M.; Xia, X.; Wang, D. The use of dextran in 3D printing for dysphagia foods: Relationships between its structure and physicochemical properties. *Food Hydrocolloids*. **2025**, *160*, 110819. https://doi.org/10.1016/j.foodhyd.2024.110819.
36. Lou, L.; Bilbao-Sainz, C.; Wood, D.; Rubinsky, B. Temperature controlled cryoprinting of food for dysphagia patients. *Innov. Food Sci. Emerg. Technol.* **2023**, *86*, 103362. https://doi.org/10.1016/j.ifset.2023.103362.
37. Li, W.; Zhang, K.; Qin, Y.; Li, M.; Li, H.; Guo, M.; Xu, T.; Sun, Q.; Ji, N.; Xie, F. Effects of sodium chloride on the textural attributes, rheological properties, microstructure, and 3D printing performance of rice starch-curdlan composite gel. *Food Chem.* **2025**, *465*, 141986. https://doi.org/10.1016/j.foodchem.2024.141986.
38. Bitencourt, B.; Guedes, J.; Saliba, A.; Sartori, A.; Torres, L.; Amaral, J.; Alencar, S.; Maniglia, B.; Augusto, P. Mineral bioaccessibility in 3D printed gels based on milk/starch/κ-carrageenan for dysphagic people. *Food Res. Int.* **2023**, *170*, 113010. https://doi.org/10.1016/j.foodres.2023.113010.
39. Carranza, T.; Guerrero, P.; Caba, K.; Etxabide, A. Texture-modified soy protein foods: 3D printing design and red cabbage effect. *Food Hydrocoll.* **2023**, *145*, 109141. https://doi.org/10.1016/j.foodhyd.2023.109141.
40. Zhang, C.; Wang, C.; Girard, M.; Therriault, D.; Heuzey, M. 3D printed protein/polysaccharide food simulant for dysphagia diet: Impact of cellulose nanocrystals. *Food Hydrocoll.* **2024**, *148*, 109455. https://doi.org/10.1016/j.foodhyd.2023.109455.
41. Zhu, Y.; Chen, L.; Zhang, X.; Meng, T.; Liu, Z.; Chitrakar, B.; He, C. 3D-Printed Pea Protein–Based Dysphagia Diet Affected by Different Hydrocolloids. *Food Bioprocess Technol.* **2023**, *17*, 1492–1506. https://doi.org/10.1007/s11947-023-03210-1.
42. Kurapkienė, A.; Vinauskienė, R.; Jasutienė, I.; Damulevičienė, G.; Knašienė, J.; Lesauskaitė, V.; Sulmont - Rossé, C.; Eisinaitė, V.; Leskauskaitė, D. One-bite-sized 3D printed finger foods, oriented to malnutrition, sarcopenia and frailty prevention in the older people. *J. Sci. Food Agric.* **2024**, *104*, 6289–6297. https://doi.org/10.1002/jsfa.13463.
43. Liu, F.; Song, X.; Bian, S.; Huang, X.; Yin, J.; Nie, S. Development of soy protein isolate gels added with Tremella polysaccharides and psyllium husk powder as 3D printing inks for people with dysphagia. *Food Funct.* **2024**, *15*, 5868–5881. https://doi.org/10.1039/d4fo00982g.
44. Qiu, R.; Wang, G.; Zhao, P.; Liu, L.; Awais, M.; Fan, B.; Huang, Y.; Tong, L.; Wang, L.; Accoroni, C. Modification of the texture of 3D printing soy protein isolate-based foods with proper nozzle sizes: A swallowing oriented strategy for dysphagia diet. *Int. J. Biol. Macromol.* **2024**, *282*, 136694. https://doi.org/10.1016/j.ijbiomac.2024.136694.
45. Kim, J.; Kim, J.; Lim, J.; Moon, K. Effects of isolated pea protein on honeyed red ginseng manufactured by 3D printing for patients with dysphagia. *LWT* **2024**, *191*, 115570. https://doi.org/10.1016/j.lwt.2023.115570.
46. Carvajal-Mena, N.; Tabilo-Munizaga, G.; Pérez-Won, M.; Herrera-Lavados, C.; Lemus-Mondaca, R.; Moreno-Osorio, L. Evaluation of physicochemical properties of starch-protein gels: Printability and postprocessing. *LWT* **2023**, *182*, 114797. https://doi.org/10.1016/j.lwt.2023.114797.
47. Zhong, Y.; Wang, B.; Lv, W.; Li, G.; Lv, Y.; Cheng, Y. Egg yolk powder-starch gel as novel ink for food 3D printing: Rheological properties, microstructure and application. *Innov. Food Sci. Emerg. Technol.* **2024**, *91*, 103545. https://doi.org/10.1016/j.ifset.2023.103545.
48. Chao, C.; Lee, J.; Kim, I.; Choi, R.; Kim, H.; Park, H. Investigation of 3D-printable chickpea-mealworm protein mixtures and their bolus rheology: A soft-textured and safe-swallowing food for the elderly. *Food Biosci.* **2023**, *54*, 102924. https://doi.org/10.1016/j.fbio.2023.102924.
49. Sartori, A.; Saliba, A.; Bitencourt, B.; Guedes, J.; Torres, L.; Alencar, S.; Augusto, P. Anthocyanin bioaccessibility and anti-inflammatory activity of a grape-based 3D printed food for dysphagia. *Innov. Food Sci. Emerg. Technol.* **2023**, *84*, 103289. https://doi.org/10.1016/j.ifset.2023.103289.
50. Guo, J.; Zhang, M.; Adhikari, B.; Ma, Y.; Luo, Z. Formulation and characterization of 3D printed chickpea protein isolate-mixed cereal dysphagia diet. *J. Agric. Food Chem.* **2023**, *253*, 127251. https://doi.org/10.1021/jf402288j.
51. Qiu, L.; Zhang, M.; Bhandari, B.; Chitrakar, B.; Chang, L. Investigation of 3D printing of apple and edible rose blends as a dysphagia food. *Food Hydrocoll.* **2023**, *135*, 108184. https://doi.org/10.1016/j.foodhyd.2022.108184.
52. Kong, D.; Zhang, M.; Mujumdar, A.; Li, J. Feasibility of hydrocolloid addition for 3D printing of Qingtuan with red bean filling as a dysphagia food. *Food Res. Int.* **2023**, *165*, 112469. https://doi.org/10.1016/j.foodres.2023.112469.
53. Qiu, L.; Zhang, M.; Adhikari, B.; Lin, J.; Luo, Z. Preparation and characterization of 3D printed texture-modified food for the elderly using mung bean protein, rose powder, and flaxseed gum. *J. Food Eng.* **2024**, *361*, 111750. https://doi.org/10.1016/j.jfoodeng.2023.111750.

54. Niu, D.; Zhang, M.; Mujumdar, A.; Li, J. Investigation of 3D printing of toddler foods with special shape and function based on fenugreek gum and flaxseed protein. *Int. J. Biol. Macromol.* **2023**, *253*, 127203. https://doi.org/10.1016/j.ijbiomac.2023.127203.
55. Wang, X.; Zhang, M.; Mujumdar, A.; Li, J. Easy-to-swallow mooncake using 3D printing: Effect of oil and hydrocolloid addition. *Food Res. Int.* **2023**, *164*, 112404. https://doi.org/10.1016/j.foodres.2022.112404.
56. Wu, J.; Zhang, M.; Devahastin, S.; Chen, H. Improving 3D printability of pumpkin pastes by addition of surimi. *J. Food Process. Preserv.* **2022**, *46*, e17127. https://doi.org/10.1111/jfpp.17127.
57. Do, Y.; Tang, T.; Zhang, M.; Mujumdar, A.; Phuhongsung, P.; Yu, D. Double-nozzle 3D-printed bean paste buns: Effect of filling ratio and microwave heating time. *J. Texture Stud.* **2023**, *54*, 671–680. https://doi.org/10.1111/jtxs.12765.
58. Huang, J.; Zhang, M.; Mujumdar, A.; Li, C. Modulation of starch structure, swallowability and digestibility of 3D printed diabetic-friendly food for the elderly by dry heating. *Int. J. Biol. Macromol.* **2024**, *264*, 130629. https://doi.org/10.1016/j.ijbiomac.2024.130629.
59. Huang, J.; Zhang, M.; Mujumdar, A.; Wang, Y.; Li, C. Improvement of 3D printing age-friendly brown rice food on rough texture, swallowability, and in vitro digestibility using fermentation properties of different probiotics. *Food Chem.* **2024**, *460*, 140701. https://doi.org/10.1016/j.foodchem.2024.140701.
60. Chao, C.; Hwang, J.; Kim, I.; Choi, R.; Kim, H.; Park, H. Coaxial 3D printing of chicken surimi incorporated with mealworm protein isolate as texture-modified food for the elderly. *J. Food Eng.* **2022**, *333*, 111151. https://doi.org/10.1016/j.jfoodeng.2022.111151.
